# Correlation between Serum Ferritin Levels and Liver Stiffness measured by Fibroscan in patients with Chronic Hepatitis C

**DOI:** 10.12669/pjms.36.3.1288

**Published:** 2020

**Authors:** Saba Latif, Quratulain Kalam, Bader Faiyaz Zuberi

**Affiliations:** 1 Dr. Saba Latif, MBBS, FCPS. Senior Registrar, Patel Hospital, Karachi, Pakistan; 2 Dr. Quratulain Kalam, MBBS, FCPS. Senior Registrar, Patel Hospital, Karachi, Pakistan; 3 Dr. Bader Faiyaz Zuberi, MBBS, FCPS. Professor of Medicine, Dow University of Health Sciences, Karachi, Pakistan

**Keywords:** Chronic hepatitis C, Ferritin, Fibroscan, Transient elastography

## Abstract

**Objective:**

To determine correlation between transient elastography values with serum ferritin and duration of infection in patients of hepatitis C.

**Methods:**

A cross-sectional study was conducted at medical units of Civil Hospital, Karachi. The study protocol was approved by the Research Evaluation Unit of College of Physician and Surgeon Pakistan (CPSP). Patients fulfilling inclusion criteria were included after taking informed consent. Serum ferritin levels were tested by standard laboratory procedures and transient elastography by fibroscan. Regression analysis was done to see correlation of ferritin with transient elastography and duration of HCV.

**Results:**

Over all 120 patients fulfilling the selection criteria were selected after informed consent. These included 68 (56.7%) male & 52 (43.3%) female. Significant differences in ferritin levels by Fibrosis stages were observed by ANOVA (df = 3; F =12.768; *p* = <0.001). Serum ferritin showed linear pattern across Fibrosis stages (F = 33.948; *p* = <0.001). Regression analysis of ferritin and duration of HCV showed significant impact on TE scores (r^2^ = 0.317).

**Conclusions:**

There is significant correlation between serum ferritin and duration of HCV with TE scores.

## INTRODUCTION

Chronic hepatitis C (CHC) is a major infectious disease which is mainly cause of morbidity worldwide in patients with liver disease, and liver transplantation.^1^ Around 2.35% of world population, i.e., approximately 160 million people are suffering with HCV.^2^ Although, its prevalence in most of the countries is 1 to 2%, but Pakistan has a high prevalence rate of 4.7%.^3^ In the last two to three decades, Chronic Hepatitis C (CHC) which progress to cirrhosis is reported in 20% of the cases; out of which 25% cases developed liver disease, hepatocellular carcinoma and such cases needed liver transplantation.^4^ Liver biopsy is the gold standard for determining the degree of liver fibrosis, but carries potentially life threatening complications. Fibroscan being a noninvasive method uses ultrasonic waves for determining liver stiffness and estimating the degree of liver fibrosis.^5^ According to a meta-analysis study, specificity and sensitivity of fibroscan in chronic patients of HCV for diagnoses of cirrhosis was estimated as 83.0% and 91.0%, respectively.^5^

Hyper-ferritinemia in CHC may reflect ongoing necroinflammatory events, and it often accompanies with iron deposits in hepatic mesenchymal cells.^6^ Raised ferritin levels play an important role of intervening the process which is associated with hepatic injury.^7^ A study demonstrates that in serum ferritin levels, the longitudinal changes are highly correlated with values of Transient Elastography (TE) acquired through fibroscan (r= 0.836, *p* <0.001).^8^ In patients who acquired the infection of HCV due to blood transfusion, the risk for cirrhosis is estimated as double.^9^ Another study results showed that fibroscan has encouragement by biochemical activity variations of liver disease in liver stiffness and CHC, and when AST is at low levels, it can underestimate the fibrosis. Therefore, it is necessary to adjust the age and AST during getting Fibroscan result so that significant accuracy could be determined.^10^ Screening with non-invasive strategies can detect the disease at early stage and intervention could be initiated.^11^,^12^

This study was conducted to determine the correlation of TE scores with duration of CHC and serum ferritin levels. This study will greatly benefit patients with hyper-ferritinemia in which presence of liver involvement would be documented. It will also help patients of CHC in determination of their liver status and thus help in better planning of their management.

## METHODS

A descriptive cross sectional study was carried out in all five medical units of Civil Hospital, Karachi, during 1^st^ June 2016 to 30^th^ November 2016. Approval of synopsis was taken from CPSP. Informed consent from the patients was taken. Patients of either sex suffering with chronic hepatitis C disease for more than six months were selected through non-probability consecutive sampling. Patients with other viral hepatitis infections, hepatic cancer, history of alcohol consumption >25g/day and patients with ascites detected by Ultrasound abdomen were excluded. The selected patients were tested for serum ferritin level by standard laboratory procedures and TE scores by fibroscan. Ten measurements of fibroscan were taken, and mean value was taken for analysis. All the procedure was done under supervision of consultant having experience of five years and above.

TE values from Fibroscan were assigned to fibrosis stages as under:

2.0-7.9 kPa = F0-F1

8.0-9.9 kPa = F2

10.0-13.9 kPa = F3

≥ 14.0 = F4

A sample size of 112 achieves 90% power to detect a difference of -0.3 between the null hypothesis correlation of 0.0 and the alternative hypothesis correlation of 0.3 using a two-sided hypothesis test with a significance level of 0.05. Sample size calculation was done using PASS 2019 software.

Data was collected through a predesigned proforma. The collected data was analyzed through SPSS version 22.0. For quantitative variables like age, disease duration, TE scores & ferritin, mean ± SD was determined, and were tested with gender by Student’s T-test. Frequency and percentages were calculated for gender and fibrosis stage and were tested by χ^2^ test. Ferritin values were tested for significance with Fibrosis score by ANOVA, test for linear model and Post-Hoc analysis were also done. Followed by regression analysis of serum ferritin with Fibrosis Score and r^2^ values were calculated. Significance was set at ≤0.05.

## RESULTS

Over all 120 patients fulfilling the selection criteria were selected after informed consent. From which 68 (56.7%) were male & 52 (43.3%) were female. Mean age of the patient was 45.8 ±14.2 years, mean hepatitis C duration was 3.8 ±1.2 years, mean TE value was 10.0 ±3.3 kPa & mean Serum Ferritin was 85.2.±35.2. No significant difference was observed in age when analyzed for gender (*p* = 0.07), while duration of HCV was found significantly increased in females (*p* = 0.041), TE values were significantly increases in females (*p* = 0.015) and serum ferritin was significantly low in females (*p* = 0.037). Details of T-Test are given in Table-I.

**Table-I:** Distribution of Age, Duration of Hepatitis C, TE Value & Serum Ferritin Student’s T-Test.

	Male n = 68	Female n = 52	Total n = 120	p value	95% Confidence Interval	

	Mean ±SD	Mean ±SD	Mean ±SD		Lower	Upper
Age (years)	43.5 ±7.5	48.6 ±19.6	45.8 ±14.2	0.070	-11.001	0.442
Duration of HCV (years)	3.6 ±1.5	4.0 ±0.7	3.8 ±1.2	0.041	-0.806	-0.017
TE value (kPa)	9.4 ±3.2	10.8 ±3.2	10.0 ±3.3	0.015	-2.623	-0.281
Serum Ferritin	91.1 ±37.4	77.5 ±31.1	85.2 ±35.3	0.037	0.859	26.249

**Fig.1:**
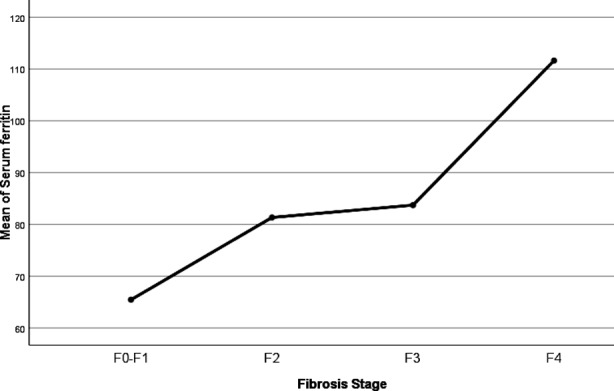
ANOVA Post-Hoc Means Plot of Serum Ferritin on Fibrosis Stage.

Serum Ferritin levels were analyzed by Fibrosis Scores by One-Way ANOVA test. Test showed significant differences in ferritin levels by Fibrosis stages (df = 3; F =12.768; *p* = <0.001). Serum ferritin showed linear pattern across Fibrosis stages (F = 33.948; *p* = <0.001). This effect is shown graphically in Fig.1. Post Hoc multiple comparisons showed significant increase in serum ferritin from F0-F1 to F2, non-significant increase from F2 to F3 & significant increase from F3 to F4. Details in Table-II.

**Table-II:** ANOVA Post Hoc multiple comparisons by Games-Howell Method.

(I) Fibrosis Stage	(J) Fibrosis Stage	Mean Difference (I-J)	p value	95% Confidence Interval	

				Lower	Upper
F0-F1	F2	-15.889*	0.002	-26.74	-5.04
	F3	-18.270*	0.027	-34.97	-1.57
	F4	-46.181*	<.001	-70.62	-21.74
F2	F0-F1	15.889*	0.002	5.04	26.74
	F3	-2.381	0.980	-18.82	14.06
	F4	-30.292*	0.009	-54.55	-6.04
F3	F0-F1	18.270*	0.027	1.57	34.97
	F2	2.381	0.980	-14.06	18.82
	F4	-27.911*	0.040	-54.94	-.88
F4	F0-F1	46.181*	<0.001	21.74	70.62
	F2	30.292*	0.009	6.04	54.55
	F3	27.911*	0.040	.88	54.94

**Fig.2:**
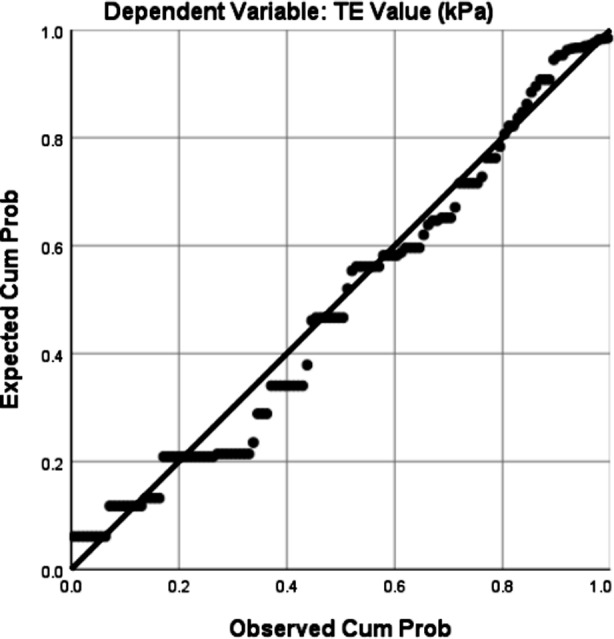
Normal P-P Plot of Regression Standardized Residual.

Regression analysis was done to see the impact of Serum Ferritin and duration of HCV (Independent Variables) on Fibrosis Score (Dependent Variable). Results showed r^2^ = 0.317 showing significant impact of the independent variables on dependent variable (Table-III). Normal P-P Plot of Regression Standardized Residual is given in Fig.2. Significance level of Serum Ferritin was p <0.001 and that of Duration of HCV was p = 0.004 (Table-IV). Thus both Serum Ferritin and Duration of HCV have significant impact on Fibrosis Score in HCV.

**Table-III:** Model summary of regression analysis.

Model Summary^b^				

Model	R	R Square	Adjusted R Square	Sth. Error of the Estimate
1	.563^a^	.317	.306	2.72986

a. Predictors: (Constant), Serum ferritin, Duration of Hep C.

b. Dependent Variable: TE Value (kPa).

**Table-IV:** Details of coefficients of regression analysis with significance.

Coefficients^a^						

Model		Unstandardized Coefficients		Standardized Coefficients	t	p value

		B	Sth. Error	Beta		
1	(Constant)	3.895	.952		4.091	<0.001
	Duration of Hep C	.619	.213	.226	2.904	0.004
	Serum ferritin	.044	.007	.477	6.145	<0.001

a. Dependent Variable: TE Value (kPa).

## DISCUSSION

TE is fast replacing invasive tests for assessment of liver fibrosis not only in adults but in pediatric population as well.^13^ Its accuracy has been well established now with several studies on this subject and to rule out significant fibrosis without invasive tests like liver biopsy.^14^,^15^ Our study showed significant correlation with cause/effect analysis of regression that duration of HCV and serum ferritin are significant contributors in fibrogenesis in HCV infection. The correlation was statistically significant our study. Documentation of ferritin as risk for fibrosis is important finding of our study with implications in patients which are at high risk to develop increased ferritin, such as patients that require repeated transfusions.^9^,^16^

Increase in fibrosis score/stage lead to many complications of cirrhosis like portal hypertension, ascites and related further complications. It is documented that CHC is a major cause of cirrhosis and hepatocellular carcinoma (HCC) around the world.^17^,^18^ In the current century, which focused more on antiviral and anti-fibrotic treatments, the demand of research and proper clinical assessment for methods of noninvasive surveillance for liver fibrosis has been significantly increased all over the world.^19^ These methods are essentials for the estimation of the advancement and deterioration of liver fibrosis.

Beveno VI consensus defined “compensated advanced chronic liver disease (cACLD) in order to better define the spectrum of advanced fibrosis and cirrhosis in asymptomatic patients, patients with TE scores between 10-15 kPa were labelled as suggestive while those with > 15 kPa were labelled as highly suggestive of cACLD”.^11^,^20^ Thus TE is an important investigation for better management of CHC. Fibrogenesis is the composite forceful interplay in different types of hepatic cell and intermediaries for preservation. Limitation of Fibroscan is that it could give falsely higher values in patients with acute liver injury and those with significant ALT elevations.^21^ Apart from Fibroscan, magnetic resonance elastography is also emerging as important non-invasive modality for assessment of liver fibrosis. Role of Ferritin is emerging as prognostic marker in cirrhosis, patients with high ferritin have been reported to have poor outcome related to hepatic insufficiency.^22^ Some studies have even shown that ferritin also predicts early mortality in decompensated cirrhosis.^23^ In study by Ricchi P et al increase in ferritin levels were shown to be responsible for raised ALT and TE scores, thus down regulation of ferritin in the early stage of fibrosis should be helpful in decreasing the inflammatory effect of ferritin.^24^ Our study also demonstrated that ferritin has a cause/effect relationship with TE scores in patients of CHC. Thus it should be checked in CHC patients specially with cACLD.

### Limitation of the Study

Our study has limitation of single center study, but we exceeded the calculated sample size to have significant effect.

## CONCLUSION

There is significant correlation with cause effect relationship of ferritin with TE scores and stages proven on regression analysis. Serum ferritin should be checked in all patients of CHC. Duration of HCV infection is another important factor related to TE scores.

### Authors’ Contribution:

**SL:** Conducted study, prepared the manuscript.

**QA:** Revised and edited manuscript, did data collection.

**BFZ:** Conceived study, did statistical analysis, gave final approval of manuscript and is responsible for integrity of research.
